# Scooping Review of Diabetes Research in Kenya from 2000 to 2020

**DOI:** 10.24248/eahrj.v8i2.784

**Published:** 2024-06-26

**Authors:** Anthony Muchai Manyara, Protus Musotsi

**Affiliations:** a School of Health and Wellbeing, University of Glasgow, Glasgow, UK; b Global Health and Ageing Research Unit, Bristol Medical School, University of Bristol, Bristol, UK; c Sentum Scientific Solutions, Kenya

## Abstract

**Background:**

The prevalence of diabetes is on the rise globally, with likely disproportionate increase in Sub-Saharan Africa. In Kenya, diabetes has been acknowledged as one of the top non-communicable diseases needing prevention and control. Research can contribute to diabetes prevention and control: however, the landscape of diabetes research in Kenya remains understudied.

**Methods:**

PubMed, MEDLINE, Scopus, PsycINFO, CINAHL, Google Scholar and ProQuest were searched for relevant articles. We included studies on humans, reporting on any type of diabetes, conducted in Kenya between 2000 to 2020.

**Results:**

From the search, 983 records were retrieved out of which 102 met the study inclusion criteria. Most studies were facility based (71%) cross sectional (65%) and descriptive (71%) conducted in Nairobi (38%) between 2013-2020 (82%), focused on diabetes control, (71%) and funded by organisations/institutions from high income countries (73%).

**Conclusion:**

Despite the recent increase in research outputs, there is still limited diabetes research being conducted in Kenya necessitating more research in the country and particularly outside Nairobi to inform prevention and control efforts. Specifically, more focus should be given to etiological and intervention studies (which use longitudinal and randomised controlled trial designs), community-based and public health research. Finally, increased local funding for diabetes research is required.

## BACKGROUND

The world is working towards achievement of Sustainable Development Goals (SDGs) with one of the targets being to reduce premature mortality, by one third, from the top four non-communicable diseases (NCDs) which include cardiovascular disease, cancer, diabetes and chronic respiratory disease.^1^ According to the International Diabetes Federation (IDF), diabetes prevalence is increasing globally with the proportion of people living with diabetes expected to increase by approximately 50% by 2045.^2^ The burden will increasingly fall on Sub-Saharan Africa (SSA) where the number of people living with diabetes will increase by >130%.^2^ Furthermore, this predicted increase maybe higher, as previous IDF projections have underestimated the diabetes burden.^3^ Economically, it has been estimated that in 2015, diabetes cost SSA 1.2% of its cumulative gross domestic product (GDP), about US$19.45 billion, and this could rise to ~2% of GDP, about $59.32 billion, in 2030.^4^ Therefore, there is an urgent need to invest in diabetes control and prevention if countries are to achieve or surpass the SDG target of reducing premature mortality from diabetes by one third by 2030.^4^

Kenya is a country in SSA which has acknowledged diabetes as one of the main NCDs and its prevalence to be escalating.^5^ Consequently, one of the objectives of the Kenyan Health Policy is to stop and reverse the increasing burden of NCDs.^6^ To inform prevention and control, several national policy guidelines have been developed. However, a recent policy analysis reported that although the Kenyan diabetes prevention and control policy documents and strategies were well aligned to international recommendations, they were based on scant local evidence.^7^ Furthermore, a recent Kenyan qualitative study involving stakeholders in NCD national policy making reported a need for more research evidence to guide practice guidelines and local interventions.^8^ Additionally, the Kenya NCD Strategic Plan 2021-2025 identifies research as one of the key pillars in achieving the NCDs reduction targets.^9^ However, the current landscape of diabetes research in Kenya remains understudied. Therefore, in this study, we explore the diabetes research conducted in Kenya to understand knowledge produced and gaps to inform future research. In particular, we aim to look at distribution of diabetes research over time, study designs, setting, focus, funding, and framing of recommendation in published studies.

## METHODS

The scoping review is an evidence synthesis method which like a systematic review requires rigor and transparency to ensure the trustworthiness of findings.^10^ It was considered more appropriate than a systematic review as the aim was to identify types of evidence in a specific area,^10^ i.e., diabetes in Kenya. The scoping review was conducted using a methodological framework proposed by Arksey and O'Malley which involves formulating a research question; identifying relevant studies; inclusion of studies; charting data; summarising and reporting results,^11^ as described below.

### Research Question Formulation and Studies Identification

The purpose of this scoping review was to review the landscape of diabetes research in Kenya. Therefore, our research question was: *what is published on diabetes in Kenya?* Studies were identified through searching eight electronic databases: PubMed, Medical Literature Analysis and Retrieval System Online (MEDLINE), EMBASE (the Excerpta Medica database), Scopus, PsycINFO, Cumulative Index to Nursing and Allied Health Literature (CINAHL), ProQuest, and Google Scholar. Two search terms (i.e., “diabetes”, “Kenya”) were combined with Boolean operator “AND”, truncated when possible to capture different versions of the terms, and adapted for each database: for example, in MEDLINE, diabet* AND Kenya* was used. The searches were conducted between March and April 2021. To complement the electronic search, reference lists of full texts were hand searched.

### Inclusion of Studies

We imported the search results from all sources into Endnote software,^12^ from where we removed duplicates. The references were then imported into Rayyan software,^13^ from where two reviewers screened them independently for eligibility. Screening for eligibility was done based on title, abstract and full text reading using inclusion criteria as follows. First, studies had to be conducted in humans of any age and not in animal models. Second, articles had to report research on any type of diabetes. Therefore, we excluded studies that used people with diabetes as convenience samples for another research area: for example, investigating HIV prevalence in people with diabetes attending a specific diabetes clinic. Third, we restricted our inclusion to the period between 2000 to 2020. The year 2000 was chosen as the start date, as it was at the beginning of the third millennium when noncommunicable diseases were acknowledged as increasing in prevalence in low and middle-income countries such as Kenya.^14^ Fourth, we included studies conducted in Kenya either entirely or in part (i.e., international studies). The differences in eligibility decisions by the two reviewers were resolved through discussion.

### Charting Data

A pre-prepared data extraction tool, piloted on ten articles, was used for the data extraction process. The tool contained the following data items: authors, year, title, journal, study design, county of study, type of diabetes, study population, study setting, study categorisation, recommendations, funding information, and the study's key findings. Data on these items was populated on Microsoft Excel sheets by the two authors independently. Any discrepancy or omission in the extracted data or item categorisation was discussed by both reviewers and agreement reached via consensus.

### Summarising and Reporting Results

All data were synthesised narratively and presented in proportions, and some descriptive data (e.g., distribution of studies by year) were presented in graphs. The study county was determined from the study setting stated in the study. For those studies conducted prior to creation of county governments in 2013, districts where the study was conducted were taken as study counties. Type of diabetes was classified as either type 1, 2 or gestational diabetes, and where the study did not explicitly state the type of diabetes studies, the age of the participants in the study was used to infer the diabetes type: type 2 being inferred for adults and type 1 for children. However, where it was not possible to infer from the ages of participants, type of diabetes was classified as unknown.

Study setting was categorised based on where it was conducted: community or facility based (health facilities). Studies that were not conducted in either of the settings, such as those using modelling approaches, were classified under others.

Further, studies were classified as either public health or non-public health. Public health studies presented evidence on diabetes prevalence, associated risk factors and correlates, knowledge and attitudes towards diabetes, and community and health promotion interventions to prevent diabetes or its complications. On the flipside, non-public health studies reported findings on diabetes control and management such as quality of care and medical interventions. Both public health and non-public health studies were classified further as descriptive, aetiological, intervention or measurement studies adapted from definitions by Milat et al.^15^ Descriptive articles presented prevalence, patterns, correlates or predictors of diabetes or diabetes-related complications. Etiological studies were epidemiological studies that investigated a causal relationship between exposure or risk factors and subsequent diabetes or diabetes complications. Further, intervention articles evaluated interventions that aimed to prevent diabetes or diabetes complications. Finally, measurement studies explored the qualities of a measurement tool such as acceptability, reliability, or validity.^15^

Study recommendations were categorised as actionable, or implications based on definitions by Goyet et al.^16^ A recommendation was classified as actionable if it specified the actor and/or the populations that should be targeted for policy or practice change; and implication if only the need for action was identified or the likely ramifications of the study findings were stated.^16^ Finally, funding was classified based on sources: high-income institutions/organisations, local institutions/organisations, combined high-income and local, and author own personal resources. We have accompanied the reporting of this scoping review with Preferred Reporting Items for Systematic reviews and Meta-Analyses extension for Scoping Reviews.^17^

## RESULTS

**Figure 1.**
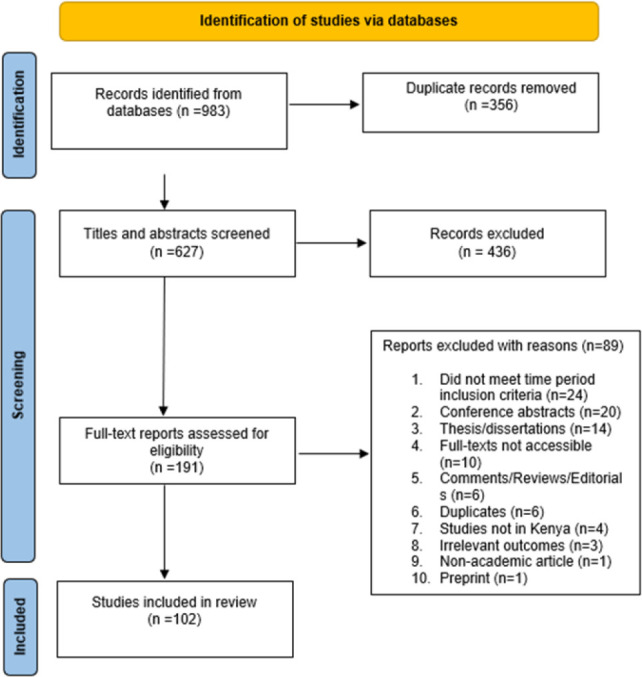
Literature Flow Diagram

Figure 1 shows the literature search flow diagram. A total of 983 records were retrieved. After removing 356 duplicates, the remaining 627 were screened for eligibility by title and abstract out of which 436 were excluded. The remaining 191 were sought for retrieval of the full articles, of which 10 could not be accessed and a further 79 were excluded with reasons. The remaining 102 articles which met the study inclusion criteria were used in the review.

**Figure 2.**
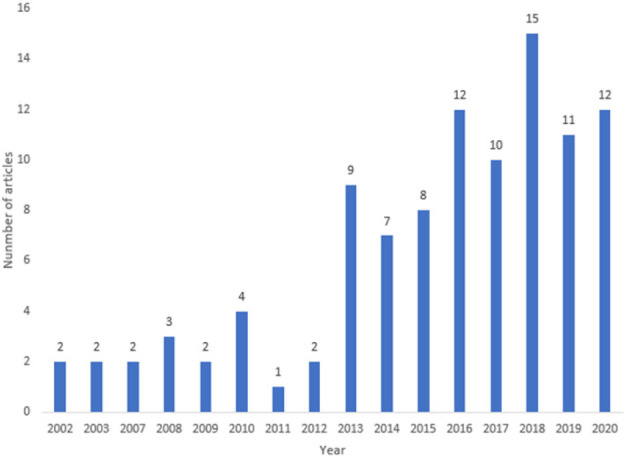
Distribution of Included Articles by Year

Figure 2 show distribution of articles by year. There was an increase in the annual diabetes research outputs: from below five a year between 2002 to 2012 to an average of 10 from 2013. Indeed, the majority of studies (n=84, 82%) were conducted between 2013 to 2020.

**Figure 3.**
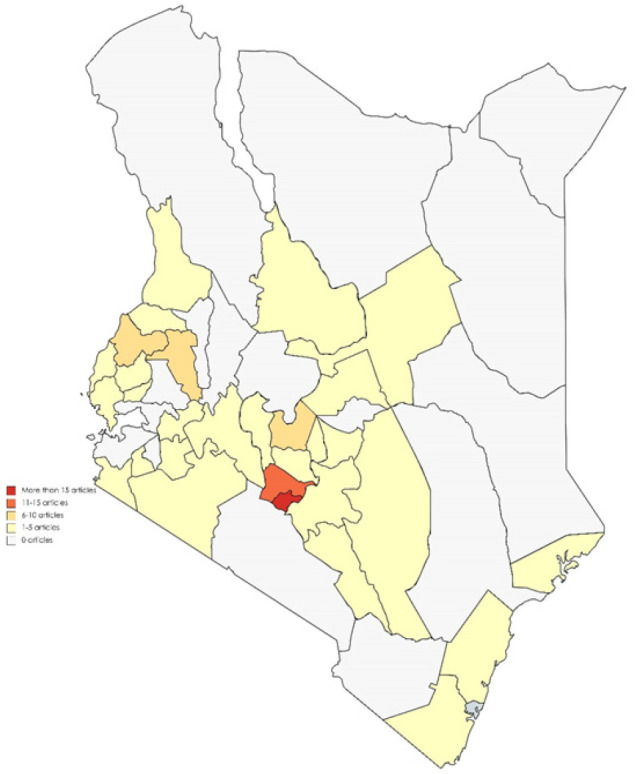
Distribution of Studies Conducted in Kenya by County Between 2000 and 2020

Figure 3 shows the distribution of studies by county. Most studies were conducted in Nairobi city county (n=39, 38%), Kiambu county (n=13, 13%), Nyeri county (n=8, 8%), Uasin Gishu County (n=7, 7%), and Bungoma county (n=6, 6%). Eight studies (8%) were reported to have nationally coverage most of which were on national policy issues not limited to any specific county hence not included on the map. Five studies (5%) also covered former regions and could not be classified to a specific county as exact study location was not specified.

Table 1 shows the study design, research area, study setting of included articles. The majority of studies used a cross-sectional design (n=66; 65%), investigated type 2 diabetes (n=72, 71%) and were facility based (n=72, 71%).

**Table 1.** Study Designs, Research Areas, and Study Setting of Included Articles

Publication characteristics	N (%)
Study design	
Case-control	1 (1)
Cohort	4 (4)
Cross sectional	66 (65)
Mixed methods	10 (9)
Others	6 (6)
Prospective	5 (5)
Qualitative	4 (4)
Quasi-experimental	1 (1)
RCT	5 (5)
Type of diabetes studied	
Gestational	2 (2)
Not specified^a^	15 (15)
Type 1	4 (4)
Type 1 and Type 2	8 (8)
Type 1, Type 2, Gestational	1 (1)
Type 2	72 (71)
Study setting	
Facility	72 (71)
Community	27 (27)
Others^b^	3 (3)

RCT: Randomised Controlled Trial

a Among the 15 studies where type of diabetes was not specified, 2 were among adult population only, hence we can infer it to be type 2 diabetes.

b Others refer to studies that analysed data and not conducted in either facilities or communities

Table 2 shows the distribution of studies by classification and type. Less than a third of the studies were public health related and the majority of studies were descriptive with very few (<25%) etiological or intervention related. One study was an analysis of diabetes prevention and control policies thus classified as both public health and non-public health.

**Table 2.** Classification and Type of Studies Included in Scooping Review

Study classification	Type of study	N (%)
Public health		29/102 (28)
	Descriptive	16/29 (55)
	Etiological	5/29 (17)
	Intervention	4/29 (14)
	Measurement	4/29 (14)
Non-public health		72/102 (71)
	Descriptive	56/72 (78)
	Intervention	13/72 (18)
	Measurement	3/72 (4)
Both public and non-public health		
	Intervention	1/102 (1)
Categorisation of all studies		
	Descriptive	72 (71)
	Etiological	5 (5)
	Measurement	7 (7)
	Intervention	18 (18)

Only 31% of the studies (32/102) had recommendations that were actionable in their abstracts, conclusion, or recommendation sections. Most of these studies (n=19, 59%) specified the target (i.e., populations to be targeted, recommended practices) while the rest (n=13, 41%) specified both the target and actors (policy makers and implementers). Over half of articles (56/102, 55%) made funding declarations: 52/56 (93%) stated they were funded, and the rest were not. Most studies (38/52, 73%) were funded by institutions, donors or organisations from high-income countries and the rest by local institutions/organisations (n=6, 12%), a combination of local and high-income sources (n=5, 10%), and from authors own personal resources (n=3, 5%).

Table 3 shows journals where more than one included article was published. Most articles (n=20, 20%) were published in African journals (East African Medical journal, Pan African Medical Journal and African Journal of Primary Health Care and Family Medicine) and only few of the articles were published in diabetes specialist international journals such as BMC Endocrine Disorders and International Journal of Diabetes and Endocrinology.

**Table 3.** Journals Involved in Multiple Publication of Same Articles

Journal	N (%)
East Africa Medical Journal	13 (13)
Pan African Medical Journal	5 (5)
Tropical Medicine and International Health	4 (4)
African Journal of Primary Health Care and	2 (2)
Family Medicine Middle East African Journal of Ophthalmology	2 (2)
BMC Endocrine Disorders	2 (2)
BMC Public Health	2 (2)
Globalization and Health	2 (2)
Global Health Action	2 (2)
International Journal of Diabetes and Endocrinology	2 (2)
Plos One	2 (2)

## DISCUSSION

We set to explore the landscape of diabetes research in Kenya between 2000 and 2020. Diabetes research had increased over the years with ≥10 articles published annually from 2016. The majority of studies were conducted in Nairobi, used a cross sectional design, investigated type 2 diabetes, explored a non-public health area (i.e., diabetes management and control) and were facility rather than community based. Furthermore, only about a third of studies had made recommendations that were actionable i.e., specified the targets and actors for policy or practice change. Finally, most studies were funded by institutions and organisations from high income countries.

The increase of diabetes research in recent years is not surprising given the increasing acknowledgment of rising NCD prevalence, including diabetes, in Kenya. This acknowledgement has been captured in policy documents such as the Kenya Health Policy 2014-2030,^18^ and the Kenya National Strategy for the Prevention and Control of Noncommunicable Diseases 2015-2020.^5^ However, the research output on diabetes remains generally low, with limited published articles in peer reviewed journals. This might be due to a number of reasons. First, it might be linked to low local diabetes research capacity (i.e. researchers, funding, institution, research environment) consistent with limited health research capacity in SSA.^19,20^ Second, it could be that some research outputs are not being published as a result of the high costs involved in publishing. Indeed, it has been established that publishing costs are prohibitive for most researchers in Kenya and SSA who rarely have grants to support dissemination efforts.^21-23^ Therefore, there is an urgent need to explore strategies that can be used to increase local research capacity and invest in knowledge translation activities such as publishing. Both could be achieved through increased funding for diabetes research.

We found that diabetes research was mainly funded by institutions and organisations from high-income countries. This has been reported in other research areas in SSA: funding agencies from high income countries are the main funders of malaria,^4^ and climate change research.^24^ Nevertheless, calls for local funding of research in SSA are long standing.^25^ Local funding may facilitate research that is in line with local priorities. A recent Kenyan qualitative study on NCDs control priority setting found that most implemented interventions were donor-driven and misalignment between donor's priorities and the country's priorities was a challenge to priority setting.^8^ Notwithstanding, intra-Africa and international collaborations have been shown to increase the visibility of research in Africa, therefore, strategies to increase research funding that enables collaboration should be explored.^25^

Despite evidence to show unaffordability of diabetes care to the majority of Kenyans,^26^ we found limited research focusing on prevention of diabetes and diabetes complications. Further, the majority of studies were conducted in health facilities rather than community settings. Additionally, most studies were descriptive with few etiological and intervention studies. This is consistent with other reviews on public health research outputs which have found descriptive studies to be the most dominant with limited intervention studies.^15,27^ The limited conduct of etiological and intervention studies could be due to the time and resource intensity associated with designing such studies i.e., longitudinal and randomised controlled trials,^28^ compared to descriptive studies. Consequently, the currently used metrics to measure the performance of researchers, which put more focus on the number of publications, coupled with lack of funding may lead more researchers to descriptive cross-sectional research,^29^ which takes less time and is cheaper. Furthermore, descriptive studies may be preferred given their less intrusive nature to participants.^27^ Nevertheless, descriptive research does not provide optimal evidence for prevention and control.^27^ Indeed, a policy analysis found that Kenyan diabetes prevention and control policy documents and strategies recommended a need for local evidence to inform tailored prevention and control measures.^7^ Therefore, to achieve the SDG target on reducing premature mortality, by one-third, from the top four NCDs including diabetes,^1^ there is an urgent need to invest in etiological and intervention research to develop tailored interventions that would reduce incidence of diabetes and diabetes complications. However, research may not be sufficient as there are already acceptable and cost-effective interventions to prevent diabetes and delay onset of complications in SSA whose implementation is a challenge to health systems already delivering suboptimal diabetes care.^30^ Consequently, there is need for strengthening of health systems to better respond to diabetes care and prevention.

The focus on type 2 diabetes by most studies is not surprising as it is the main form of diabetes accounting for 90 to 95% of cases globally.^31^ Similarly, conduct of the majority of studies in Nairobi city county was expected given that it is Kenya's largest urban setting and a national study shows that diabetes prevalence is higher in urban compared to rural settings.^32^ Nevertheless, it was surprising that few studies were found from other urban counties in particular Mombasa and Kisumu cities which necessitates more diabetes research in these settings which may have a high burden.

Finally, most articles were published in general medical and public health journals and one in five articles were published in local journals. Publishing in local journals could be common for two reasons. First, local journals tend to be cheaper: charging about US$100 to 200,^33,34^ for article processing charges compared to an average of about US$ 2000,^35,36^ in journals based in high-income countries. Second, it could be that local journals provide the best platform for local knowledge dissemination and exchange.^37^ However, recent evidence suggests that articles published in African journals have a lower visibility compared to those published in international journals.^25^ Taken together, these findings imply that readers with interest in Kenyan diabetes research are more likely to find such research in local rather than international journals and in general and medical rather than diabetes specialist journals. Furthermore, given usefulness of local journals, there is need to improve the quality of such journals to increase visibility and quality of published articles.^25^

### Limitations

Despite extensive search of peer reviewed publications, we were not able to search and include grey literature due to time constraints which may have led to an underestimation of diabetes research. Additionally, we limited our review to 2000, hence likely to have missed out on articles published before this period. However, it is less likely that much was published before this period considering the trend observed over the years and the lack of any article published in 2000 to 2001 meeting our eligibility criteria.

## CONCLUSION

The review has described the diabetes research landscape in Kenya. Most of the studies are: descriptive and use cross-sectional study designs; focus on type 2 diabetes; investigate diabetes control rather than prevention; facility-based rather than community based; conducted in Nairobi; and are funded by organisations/institutions from high-income countries. From the review, it is evident that although diabetes research outputs have increased in the last decade, there is still limited diabetes research being conducted in Kenya. In particular, there is an urgent need for increase in etiological and intervention studies (i.e., use of longitudinal and randomised controlled trial designs), community-based and public health research to inform local diabetes prevention and control efforts. Furthermore, there is need for more evidence in other counties other than Nairobi. Finally, more local funding is required to facilitate increase in diabetes research.
